# Antifungal Activity of Fused Mannich Ketones Triggers an Oxidative Stress Response and Is Cap1-Dependent in *Candida albicans*

**DOI:** 10.1371/journal.pone.0062142

**Published:** 2013-04-30

**Authors:** Tristan Rossignol, Béla Kocsis, Orsolya Bouquet, Ildikó Kustos, Ferenc Kilár, Adrien Nyul, Péter B. Jakus, Kshitij Rajbhandari, László Prókai, Christophe d’Enfert, Tamás Lóránd

**Affiliations:** 1 Institut Pasteur, Unité Biologie et Pathogénicité Fongiques, Département Génomes et Génétique, Paris, France; 2 INRA, USC 2019, Paris, France; 3 Department of Medical Microbiology and Immunology, Faculty of Medicine, University of Pécs, Pécs, Hungary; 4 Institute of Bioanalysis, Faculty of Medicine, University of Pécs, Pécs, Hungary; 5 Department of Microbiology, Alder Hey Children`s NHS Foundation Trust, Liverpool, United Kingdom; 6 Department of Biochemistry and Medical Chemistry, Faculty of Medicine, University of Pécs, Pécs, Hungary; 7 Department of Molecular Biology and Immunology, University of North Texas Health Science Center, Fort Worth, Texas, United States of America; Instituto de Salud Carlos III, Spain

## Abstract

We investigated the antifungal activity of fused Mannich ketone (FMK) congeners and two of their aminoalcohol derivatives. In particular, FMKs with five-membered saturated rings were shown to have minimum inhibitory concentration (MIC_90_s) ranging from 0.8 to 6 µg/mL toward *C. albicans* and the closely related *C. parapsilosis* and *C. krusei* while having reduced efficacy toward *C. glabrata* and almost no efficacy against *Aspergillus sp*. Transcript profiling of *C. albicans* cells exposed for 30 or 60 min to 2-(morpholinomethyl)-1-indanone, a representative FMK with a five-membered saturated ring, revealed a transcriptional response typical of oxidative stress and similar to that of a *C. albicans* Cap1 transcriptional activator. Consistently, *C. albicans* lacking the *CAP1* gene was hypersensitive to this FMK, while *C. albicans* strains overexpressing *CAP1* had decreased sensitivity to 2-(morpholinomethyl)-1-indanone. Quantitative structure–activity relationship studies revealed a correlation of antifungal potency and the energy of the lowest unoccupied molecular orbital of FMKs and unsaturated Mannich ketones thereby implicating redox cycling-mediated oxidative stress as a mechanism of action. This conclusion was further supported by the loss of antifungal activity upon conversion of representative FMKs to aminoalcohols that were unable to participate in redox cycles.

## Introduction

The number of invasive fungal infections has dramatically increased over the recent years causing morbidity and mortality of immunocompromised patients [1]. In particular, patients after transplantation, under corticosteroid therapy, burn patients, drug abuser and neonates are in a high risk group for systemic fungal infection. Current treatment of invasive fungal infections relies mainly on three families of antifungal compounds: azoles that target membrane ergosterol biosynthesis, echinocandins that target cell wall beta-1,3-glucan biosynthesis and polyenes that target membrane ergosterol [2]. Nevertheless, high mortality rates for invasive candidiasis (30–50%) and invasive aspergillosis (50–90%) are observed despite this antifungal arsenal [1]. Therefore, the discovery and development of new, efficient antifungal agents are important endeavors [2].

Mannich ketones (MKs) are versatile starting materials for synthetic organic and medicinal chemistry [3]. Their decomposition in an elimination reaction yields very reactive vinyl ketones as possible intermediates for ring closure in the synthesis of heterocycles. MKs can also be used as prodrugs [4], [5]. Furthermore, unsaturated MKs are efficient alkylating agents for thiol enzymes [6], [7] and their water solubility, especially in the case of the aminoketones, makes them good model compounds for antimicrobial investigations.

Previously, we have reported the synthesis and structure verification of two families of MKs: a set of 23 unsaturated MKs [8] and another one represented by 21 fused MKs (FMKs) [9]. Their reduction yielded 11 compounds as the corresponding aminoalcohols [10]. Interestingly, compounds of these three families showed antibacterial activity [8]–[10]. This activity could be correlated, to some extent, with the ability of the unsaturated and fused MKs to deplete thiols. However, several compounds with efficient antibacterial activity did not cause thiol depletion, suggesting that there were other chemical determinants of the antibacterial efficacy of MKs beside thiol depletion [11], [12]. The antifungal activity of MKs was also investigated. In particular, sixteen compounds among the unsaturated MKs and three compounds from the family of aminoalcohols showed antifungal activity towards reference *Candida* strains and some clinical isolates [13]. However, the antifungal mode-of-action of these compounds has remained unknown.

**Figure 1:**
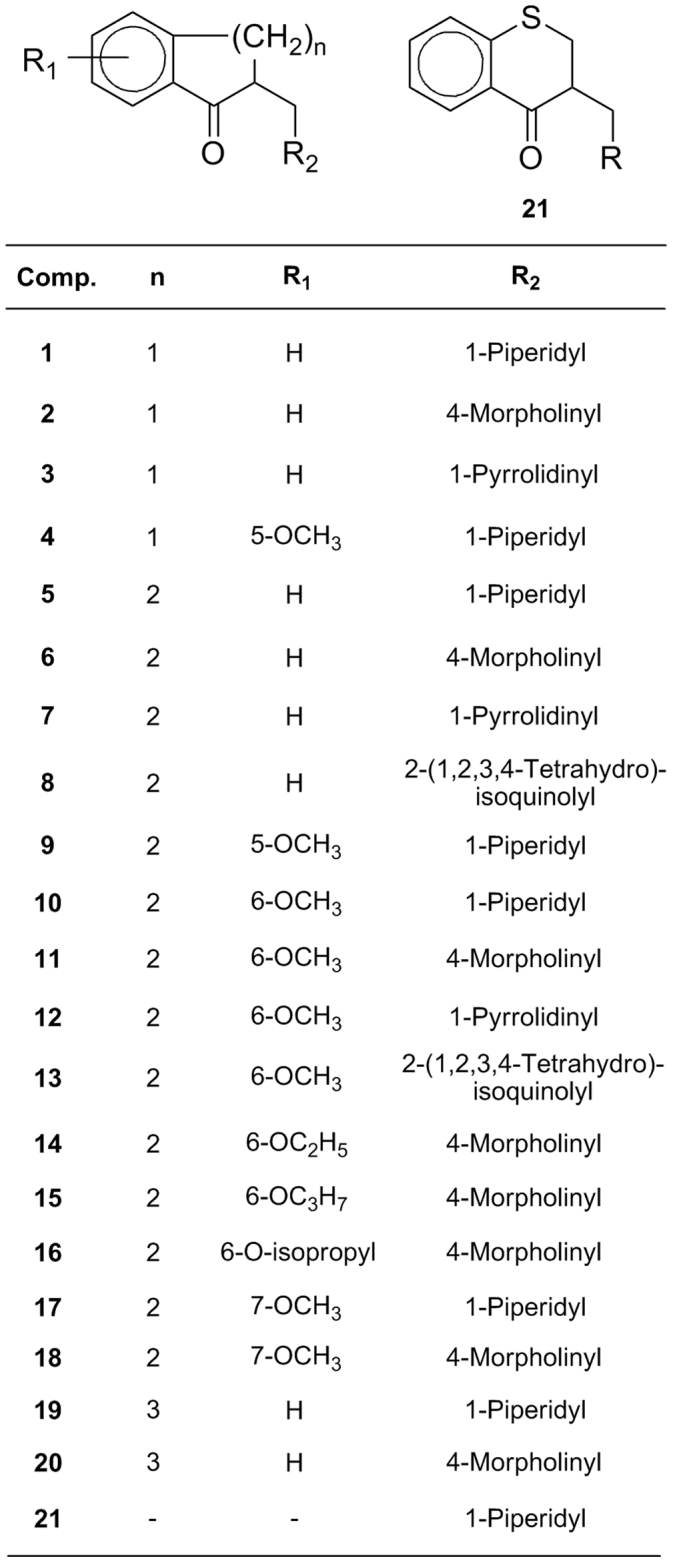
Chemical structures of the fused Mannich ketones investigated.

**Figure 2:**
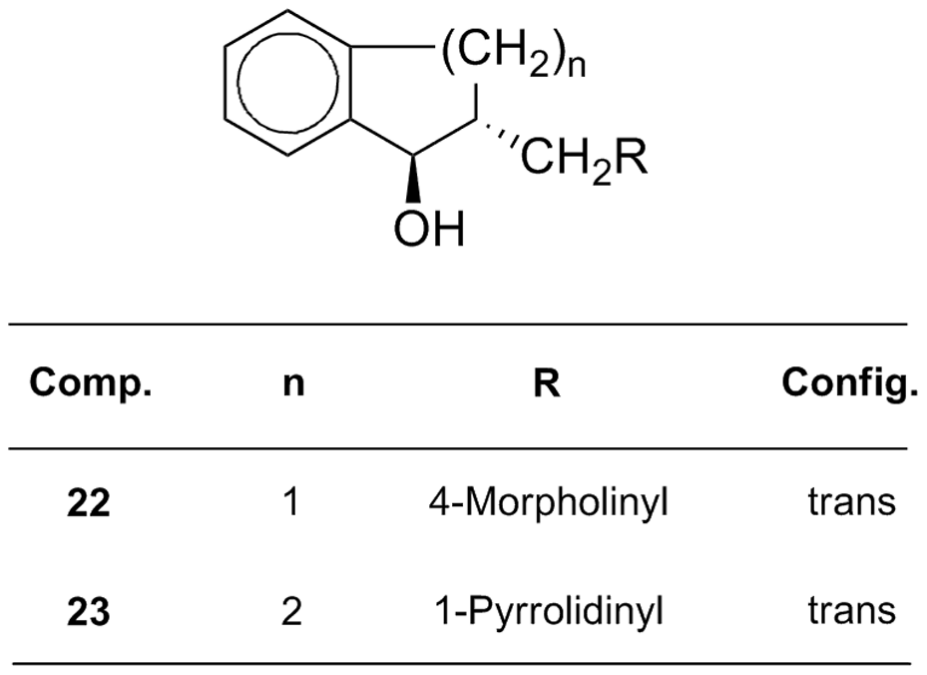
Chemical structure of the aminoalcohols investigated.

Here we report the characterization of the antifungal activity of a family of FMKs (Fig. 1) and their two aminoalcohol congeners (Fig. 2), along with a preliminary investigation of their mode of action in *Candida albicans* using transcript profiling, analysis of *C. albicans* mutants and quantitative structure–activity relationship (QSAR) analysis. Our combined approach strongly suggests that FMKs trigger oxidative damage in *C. albicans*, which contributes to their antifungal activity.

## Results

### Fused Mannich Ketones (FMK) show Antifungal Activity Towards several pathogenic yeasts

In a previous study [9], we have reported the synthesis of 21 FMKs (Fig. 1) and their aminoalcohol congeners (Fig. 2). Here, we focus on their antifungal activity against several fungal pathogens of humans; namely *Candida albicans*, *Candida krusei* and *Candida parapsilosis*. These fungi belong to the CTG clade of hemiascomycetous yeasts, *Candida glabrata* (a hemiascomycetous yeast closely related to *Saccharomyces cerevisiae*) and the filamentous ascomycete *Aspergillus fumigatus.* We also evaluated the antifungal activity of these compounds towards a *Saccharomyces sp.* clinical isolate.

MIC_90_s for each of the FMKs are given in Table 1. Noticeably, most compounds, except compound **2** (2-(morpholinomethyl)-1-indanone), had MIC_90_s above 50 µg/mL, when assayed towards *Aspergillus sp.* Moreover, compound **5**, **7** and the alkoxy-substituted six-membered compounds **9–18** and the two amino alcohols **22–23** showed weak potency with MIC_90_s from 6.25 µg/mL to 200 µg/mL (Table 1) across all species tested. In contrast, compounds **1–4**, **6**, **8** and **19–21** displayed appreciable antifungal activity against the different yeast species (MIC_90_s ranging from 0.8 to 12.5 µg/mL). However, MIC_90_s were significantly higher for *C. glabrata* than for other yeast species which was in agreement with previous observations showing decreased sensitivity of *C. glabrata* to antifungals such as azoles and the antifungal peptide ApoEdpL-W [1], [14], [15], [16], [17]. Compound **6** and compounds with seven-membered saturated rings (**19** and **20**) showed reduced activity towards *C. parapsilosis*. Overall, MKs with a five-membered saturated ring (compounds **1–4)** exerted the most potent antifungal effect against all yeast strains tested, with compound **2** being the only one exerting activity toward *Aspergillus sp.* Taken together, these results indicated that a subset of the tested FMKs have antifungal activity against pathogenic yeasts, with the best compounds being FMKs **1**–**4** that contain a five-membered saturated ring.

**Table 1:** MIC90s of fused Mannich ketones and aminoalcohols towards *Candida*, *Saccharomyces* and *Aspergillus* species.

Comp.	MIC_90_ (µg/mL)					
	*Candida albicans*	*Candida glabrata*	*Candida krusei*	*Candida parapsilosis*	*Saccharomyces sp.*	*Aspergillus sp.*
**1**	3.125	6.25	3.125	1.56	0.8	100
**2**	6.25	6.25	3.125	3.125	1.56	6.25
**3**	0.8	6.25	3.125	3.125	1.56	50
**4**	3.125	6.25	1.56	6.25	3.125	50
**5**	12.5	100	12.5	12.5	12.5	100
**6**	3.125	25	3.125	12.5	6.25	50
**7**	12.5	50	25	12.5	25	100
**8**	6.25	12.5	3.125	6.25	6.25	50
**9**	12.5	200	25	25	100	50
**10**	50	200	100	100	50	200
**11**	25	100	50	50	25	100
**12**	100	200	200	200	200	>200
**13**	25	100	25	25	12.5	200
**14**	12.5	25	25	25	25	50
**15**	25	50	25	25	6.25	25
**16**	25	50	50	50	25	100
**17**	6.25	50	12.5	25	50	100
**18**	12.5	50	50	50	25	200
**19**	6.25	12.5	6.25	25	6.25	50
**20**	12.5	12.5	1.56	25	3.125	100
**21**	1.56	6.25	0.8	0.8	1.56	50
**22**	>200	>200	>200	>200	>200	>200
**23**	>200	>200	>200	>200	>200	>200
**A** a	0.4	0.4	0.4	0.4	0.8	3.125

a Amphotericin B was used as a standard.

We have previously shown that, according to the Hodge and Sterner Scale [18], compounds **1**, **3** and **4** have acceptable toxicity to HeLa cells (IC_50_ around 15, 12 and 14 µg/mL, respectively), while compound **2** showed a higher toxicity (IC_50_ = 2.5 µg/mL) [9]. As compound **2** has a broader spectrum of antifungal activity (i.e., it is the only one among the tested FMKs with activity against *Aspergillus sp.*), it was selected for further in vivo investigation. Compound **2** was given to BALB/c mice intraperitoneally and the LD_50_ value was calculated from the dose–effect curve using the Lichfield–Wilcoxon graphic method [19], [20]. An LD_50_ of 450 mg i.p./body weight kg (1.68 mM. i.p./body weight kg) was obtained, indicating moderate toxicity according to the Hodge and Sterner Scale [18]. Practically this value is at the borderline between moderate and weak toxicity.

### Quantitative Structure–activity Relationships

**Figure 3:**
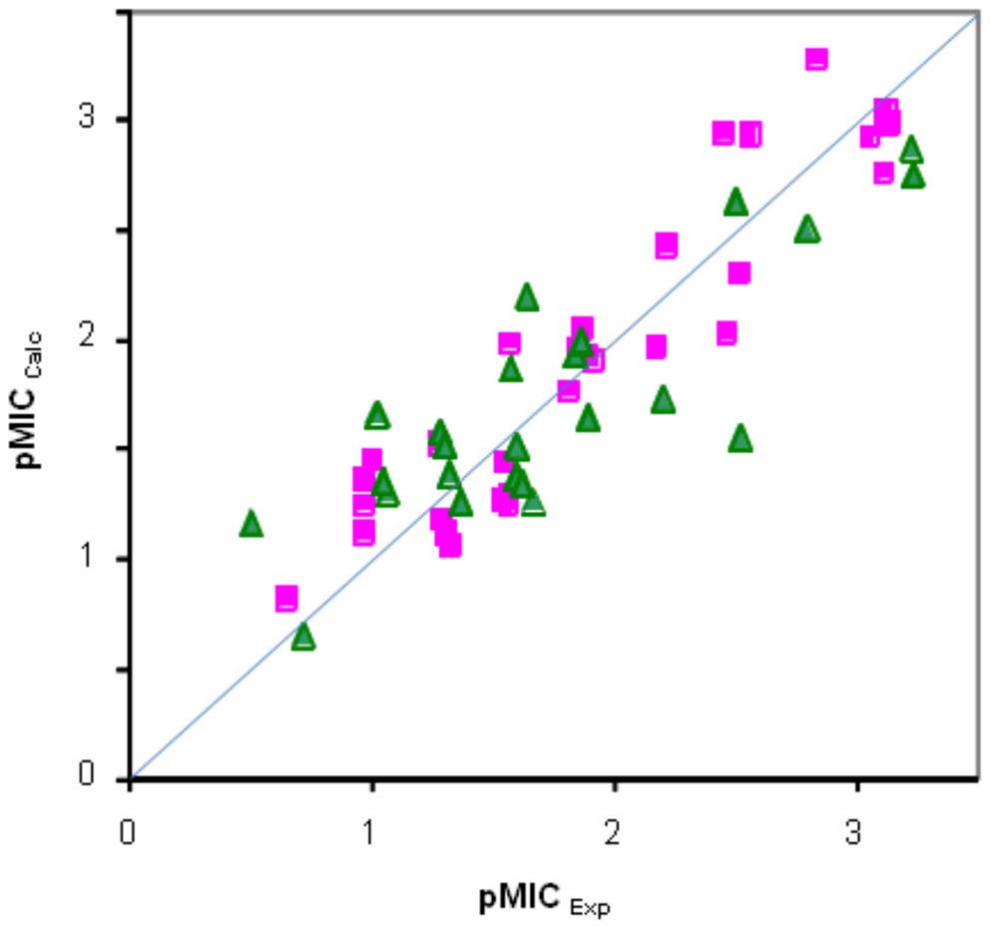
QSAR analysis of Mannich ketones for antifungal activity towards *Candida albicans*. Comparison of the experimental and calculated negative logarithms of the minimum inhibitory concentration from QSAR (pMIC_Exp_ and pMIC_Calc_) of Mannich ketones in *C. albicans* was based on the equation considering three descriptors: energy of the lowest unoccupied molecular orbital (LUMO, eV) solvent-accessible surface area (SASE, Å^2^), and ionization potential (IP, eV) (Table 2). Green triangles: Fused Mannich ketones reported here; Magenta squares: unsaturated cyclic Mannich ketones and aminoalcohols reported earlier [13].

We performed a QSAR study to correlate the potency to inhibit the growth of the various yeast strains (*C. albicans*, *C. glabrata*, *C. krusei*, *C. parapsilosis* and *Saccharomyces sp.*) with the molecular properties of FMKs (Table 1), unsaturated cyclic MKs [13] and aminoalcohols [13]. The best three- and two-parameter equations that were obtained from this analysis are listed in Table 2. As shown by the representative example in Fig. 3, data for the unsaturated cyclic MKs and the FMKs could be merged to give common QSARs. The universal descriptor found to correlate with pMIC was the energy of the lowest unoccupied molecular orbital (LUMO, eV). In addition, a shape index (first-order basic κ-type, SI_κ1_) was represented for all *Candida* strains. Solvent accessible surface area (SASE, Å^2^) and dielectric energy (E_De_, kcal/mol) were also common descriptors among the best QSARs considering two and three descriptors. Ionization potential (IP, eV), steric energy (E_St_, kcal/mol), the energy of the highest occupied molecular orbital (HOMO, eV), the valence connectivity index (zero-order, standard VC_0_) and molar refractivity (MR) were represented in single equations. The additional descriptors (SI_κ1_, SASE, IP, E_St_, HOMO, VC_0_ and MR) may indicate the role of specific mechanisms (e.g., binding to protein targets yet to be identified) not directly related to or complementing the antifungal activity of unsaturated MKs and FMKs.

**Table 2:** QSAR models for antifungal potency of Mannich ketones.^a^

Fungal Strain	Best 3-descriptor equation^a^	Best 2-descriptor equation^a^
*C. albicans*	pMIC = 2.68*LUMO +0.006*SASE +0.92*IP –7.78R^2^ = 0.81	pMIC = 2.57*LUMO +0.09*SI_κ1_+1.23R^2^ = 0.78
*C. glabrata*	pMIC = 2.46312*LUMO –0.17*Polar +0.38*SI_κ1_+2.80R^2^ = 0.68	pMIC = 2.68*LUMO –2.67*E_De_ +2.08R^2^ = 0.64
*C. krusei*	pMIC = 2.30*LUMO −1.12*HOMO +0.007* **SASE** –9.83R^2^ = 0.63	pMIC = 2.17*LUMO +0.10*SI_κ1_+1.05R^2^ = 0.58
*C. parapsilosis*	pMIC = 2.43*LUMO+0.005*SASE+0.03*E_St_ +0.81R^2^ = 0.74	pMIC = 2.47*LUMO +0.08* **SI_κ1_**+1.40R^2^ = 0.71
*Saccharomyces sp.*	pMIC = 2.70*LUMO –0.12*MR+0.86*VC_0_+2.07R^2^ = 0.65	pMIC = 2.83*LUMO –3.02*E_De_ +1.47R^2^ = 0.61

a Includes unsaturated cyclic Mannich ketones and aminoalcohols reported in Kocsis *et al.* [13].

### Gene Expression Profiling of *Candida albicans* SC5314 Exposed to Fused Mannich Ketones

To investigate the antifungal mode of action of FMKs, we performed transcriptional profiling of *C. albicans* SC5314 cells exposed to compound **2–** the only compound having broad antifungal activity in our set of FMKs. Compound **2** showed an MIC_90_ of 6.25 µg/mL towards *C. albicans* strain SC5314 grown in SD medium, similar to the MIC_90_ defined for strain ATCC90028 (Table 3). Hence, exponentially growing *C. albicans* SC5314 cells were exposed to compound **2** at 6.25 µg/mL for 30–60 min and the levels of expression of 6852 putative ORFs in the treated and untreated cells were compared using microarrays (see Materials and Methods for details). Following 30 min of exposure, we identified 138 genes up-regulated in response to compound **2** while 40 genes were down-regulated (Supplemental Table S1). After 60 min, 111 genes were identified as up-regulated and 84 as down-regulated (Supplemental Table S1). Overall, 56 up-regulated genes and 8 down-regulated genes were shared by the 30- and 60-min transcript profiles.

**Table 3:** Fungal strains used in this study.

Strains	Species	Description	Genotype	Reference
ATCC 90028	*C. albicans*	Standard strain		
ATCC 3916	*C. glabrata*	Standard strain		
ATCC 30068	*C. krusei*	Standard strain		
ATCC 22019	*C. parapsilosis*	Standard strain		
8897/2000 Pécs	*Saccharomyces sp.*	Clinical sample		
10159/2000 Pécs	*Aspergillus sp.*	Clinical sample		
SC5314	*C. albicans*	Sequenced strain		[61]
CAI4	*C. albicans*		*ura3*▵::λ·*imm434/ura3*▵:: λ·*imm434*	[62]
BWP17	*C. albicans*		*ura3*▵:: λ·*imm434/ura3*▵:: λ·*imm434 his1*::*hisG/his1*::*hisG arg4*::*hisG/arg4*::*hisG*	[63]
CNC13	*C. albicans*	Deleted for *HOG1* (orf19.895)	*ura3*▵:: λ·*imm434/ura3*▵:: λ·*imm434 his1▵::hisG/his1▵::hisG hog1▵::hisG/hog1▵::hisG-URA3-hisG*	[29]
DSY3410-1	*C. albicans*	Deleted for *MNL1* (orf19.6121)	BWP17*mnl1::tn7-UAU1/mnl1::tn7-URA3*	[63]
DSY1691	*C. albicans*	Deleted for *CTA4* (orf19.7374)	*ura3*▵:: λ·*imm434/ura3*▵:: λ·*imm434 cta4*Δ : : *hisG/cta4*Δ : : *hisG-URA3-hisG*	[64]
CJD21	*C. albicans*	Deleted for *CAP1* (orf19.1623)	*ura3*▵:: λ·*imm434/ura3*▵:: λ·*imm434 cap1▵::hisG/cap1▵::hisG*	[24]
Strains	*Species*	Description	*Genotype*	Reference
CEC3490	*C. albicans*	Overexpressing *CAP1*	*ura3*▵:: λ·*imm434/ura3*▵:: λ·*imm434 his1▵::hisG/HIS1 arg4▵::hisG/ARG4 RPS1/rps1:: CIp10-URA3-P_PCK1_-CAP1-TAPtag*	This study

In order to get further insights on the transcriptional responses of cells exposed to compound **2**, up-regulated and down-regulated genes were queried according to the *C. albicans* gene ontology [21], [22]. Noticeably, a significant enrichment for genes associated with oxidative stress was observed among the genes up-regulated at 30 min (*P*-value = 3.23·10^−9^) or 60 min (*P*-value = 7.59·10^−5^). Furthermore, the set of genes down-regulated at 30 min was enriched for genes involved in fatty acid biosynthesis (*P*-value = 1.2·10^−4^), whereas the set of genes down-regulated at 60 min was enriched for genes involved in monocarboxylic acid metabolism (*P*-value = 3.97·10^−6^), including genes involved in the glyoxylate cycle and fatty acid metabolism. This suggested an oxidative stress signal and a down regulation of fatty acid metabolic pathways in response to compound **2**.

Additionally, we noted that a large number of the genes up-regulated at both time-points had been previously annotated as “Cap1 regulated” (Table 4; [23]). Cap1 is a transcription factor central to *C. albicans* oxidative stress response [24]. Wang *et al.* [25] have identified *C. albicans* genes that respond to oxidative stress (H_2_O_2_) and defined those regulated in a Cap1-dependent manner. Genes induced by compound **2** overlapped with the H_2_O_2_-induced genes defined by Wang *et al.* (18 out of 56 genes; [25]), and the majority of the overlapping genes were Cap1 regulated (Table 4). Strikingly, 35 of the 56 genes induced by compound **2** have been characterized as being bound by Cap1 in their promoter region (Table 4; [26]). Moreover, 50 of the 193 genes up-regulated in response to compound **2** at any one of the two time points were part of the 89 Cap1 targets identified by Znaidi *et al.* [26]. These results suggested that compound **2** triggered oxidative damage and activation of the Cap1 regulon in *C. albicans*.

**Table 4:** *C. albicans* genes showing increased expression upon 30 min and 60 min exposure to compound 2.

Systematic name	Gene name		Ratio 30 min		*P*-value 30 min		Ratio 60 min		*P*-value 60 min		Benomyl^1^		Cap1-dependent^2^		H_2_0_2_ 3	Benomyl^4^	Benomyl Cap1-dependent^5^	Cap1-regulated^6^		Short description	
orf19.113	CIP1		215.4		1.07E-03		95.99		6.54E-06				▪		▪	▪	▪	▪		Possible oxidoreductase	
orf19.3131	OYE32		61.7		9.09E-04		30.13		2.87E-06		▪		▪		▪	▪	▪	▪		NAD(P)H oxidoreductase family protein	
orf19.5604	MDR1		24.98		2.01E-03		42.8		1.16E-06						▪	▪	▪	▪		Plasma membrane multidrug efflux pump	
orf19.3139			23.76		1.36E-04		3.842		5.47E-08		▪		▪		▪	▪	▪			Hap43p-repressed gene	
orf19.2285			17.63		4.76E-03		14.65		3.35E-05		▪					▪	▪	▪		Increased transcription is observed upon benomyl treatment	
orf19.251			16.43		4.27E-08		2.994		9.48E-06				▪		▪	▪	▪	▪		ThiJ/PfpI protein	
orf19.5285	PST3		15.56		9.92E-03		6.958		1.14E-07						▪			▪		Putative flavodoxin	
orf19.3443	OYE2		11.7		8.98E-04		6.748		4.21E-07									▪		Putative NAPDH dehydrogenase	
orf19.1149	MRF1		11.42		4.92E-03		6.223		1.56E-07		▪		▪		▪	▪		▪		Putative mitochondrial respiratory protein	
orf19.2262			11.28		2.38E-03		6.234		5.68E-10		▪		▪		▪	▪	▪	▪		Protein similar to quinone oxidoreductases	
orf19.6898			9.273		3.03E-03		9.889		7.11E-10		▪					▪	▪	▪		Predicted ORF	
orf19.3121	GST1		9.049		1.81E-02		1.588		4.03E-02		▪					▪	▪	▪		Putative glutathione S-transferase	
orf19.7042			8.096		2.48E-03		7.62		1.99E-03		▪					▪	▪	▪		Increased transcription is observed upon benomyl treatment	
orf19.1763	IFR1		6.616		2.78E-02		3.001		1.08E-04						▪	▪	▪	▪		Predicted ORF	
orf19.5286	YCP4		6.288		6.19E-03		3.602		4.10E-06											Putative flavodoxin	
orf19.2396	IFR2		5.712		1.06E-04		3.128		2.11E-07		▪		▪		▪	▪	▪	▪		Zinc-binding dehydrogenase	
orf19.5517			5.633		1.67E-04		1.952		4.42E-06		▪		▪		▪	▪	▪	▪		Similar to alcohol dehydrogenases	
orf19.7306			5.539		3.30E-02		3.896		1.62E-06		▪					▪				Aldo-keto reductase family protein	
orf19.847	YIM1		5.318		5.94E-04		2.895		3.70E-05		▪					▪	▪	▪		Protein similar to protease of mitochondrial inner membrane	
orf19.1340			5.312		4.93E-05		1.582		1.38E-03							▪	▪			Putative aldose reductase	
orf19.1237	ARO9		5.082		2.22E-02		2.141		1.98E-04							▪				Aromatic transaminase of the Ehrlich fusel oil pathway of aromatic alcohol biosynthesis	
orf19.3150	GRE2		5.012		4.43E-03		3.405		3.53E-06		▪					▪		▪		Putative reductase; benomyl- induced	
orf19.7531			4.633		1.87E-02		2.131		1.26E-06		▪					▪				Putative protein of unknown function	
orf19.2693	GST2		4.453		1.16E-03		3.151		8.04E-06		▪					▪		▪		Putative glutathione S transferase	
orf19.1048	IFD6		3.835		7.52E-03		2.548		1.73E-02							▪		▪		Aldo-keto reductase family member	
orf19.1868	RNR22	3.83		4.09E-02		1.825		3.42E-02								▪			▪		Putative ribonucleoside diphosphate reductase
orf19.3234	OYE22	3.777		2.30E-02		1.713		7.88E-04								▪	▪		▪		Putative NADPH dehydrogenase
orf19.3122	ARR3	3.609		1.75E-06		2.951		1.19E-08		▪				▪		▪	▪		▪		Protein not essential for viability
orf19.4309	GRP2	3.59		4.39E-03		1.756		6.74E-07		▪		▪		▪		▪			▪		Methylglyoxal reductase
orf19.1167		3.29		4.06E-02		2.175		8.90E-03								▪					*S. cerevisiae* ortholog Jlp1p
orf19.1162		3.22		1.09E-02		2.398		8.66E-07		▪						▪	▪				Predicted ORF
orf19.7611	TRX1	3.179		2.38E-03		1.543		5.88E-05		▪									▪		Thioredoxin, involved in response to reactive oxygen species
orf19.6586		3.138		3.50E-02		2.88		3.02E-05		▪						▪	▪		▪		Late-stage biofilm-induced gene
orf19.2500		2.998		2.26E-03		1.625		3.08E-04													
orf19.6059	TTR1	2.908		1.00E-03		2.024		1.51E-04		▪				▪		▪	▪				Putative glutaredoxin
orf19.2461	PRN4	2.8		4.61E-03		2.263		5.67E-06		▪						▪					Protein with similarity to pirins
orf19.4449		2.766		4.93E-03		1.655		3.31E-03								▪	▪		▪		*S. cerevisiae* ortholog Ccs1p has superoxide dismutase copper chaperone activity
orf19.1027	PDR16	2.456		4.71E-02		2.278		2.58E-05											▪		Phosphatidylinositol transfer protein
orf19.2369.1	ATX1	2.389		3.37E-03		1.608		1.63E-04								▪					Putative cytosolic copper metallochaperone
orf19.2825		2.356		7.77E-05		1.822		4.46E-05											▪		Putative cytosolic Fe-S protein assembly protein
orf19.2862	RIB1	2.216		1.16E-04		1.653		1.22E-04								▪	▪		▪		Putative GTP cyclohydrolase II; enzyme of riboflavin biosynthesis
orf19.5258		2.156		4.63E-03		1.681		1.05E-04								▪			▪		Predicted ORF
orf19.1623	CAP1	2.11		6.74E-04		1.738		2.30E-04						▪		▪	▪		▪		Transcription factor, AP-1 bZIP family; role in oxidative stress response
orf19.2202		2.093		4.55E-02		1.508		2.51E-02								▪					Predicted ORF
orf19.2463	PRN2	1.993		6.57E-03		1.851		1.93E-04								▪					Protein similar to pirin; Hap43p-repressed gene
orf19.5860		1.984		4.95E-02		1.792		9.01E-04													Predicted ORF
orf19.5784	AMO1	1.911		1.16E-02		1.699		3.29E-05													Putative peroxisomal copper amine oxidase
orf19.344		1.885		1.46E-04		1.803		1.80E-02								▪	▪		▪		Predicted ORF
orf19.1724		1.878		2.09E-03		1.975		9.36E-06													Protein of unknown function
orf19.6478	YCF1	1.861		3.96E-03		1.648		8.07E-05				▪		▪					▪		Putative glutathione S-conjugate transporter
orf19.3448		1.812		1.08E-02		1.528		2.59E-04													Predicted ORF
orf19.3130		1.762		4.42E-03		1.883		1.18E-05								▪	▪				Predicted ORF
orf19.5282		1.73		4.96E-04		1.527		8.35E-03											▪		Hap43p-repressed gene
orf19.2462	PRN3	1.555		2.49E-02		1.972		4.43E-05								▪	▪				Protein similar to pirin
orf19.3395		1.544		6.52E-03		1.615		2.89E-02						▪		▪	▪		▪		Predicted membrane transporter
orf19.4757	NAR1	1.518		3.72E-03		1.851		1.02E-03				▪		▪		▪					Putative cytosolic iron-sulfur (FeS) protein

1 Annotated as benomyl regulated in CGD database.

2 Annotated as Cap1 dependent regulation in CGD database.

3 Induced by H_2_O_2_ according to Wang *et al.* [25].

4 Induced by Benomyl according to Znaidi *et al.* [26].

5 Induced by benomyl in a Cap1 dependent manner according to Znaidi *et al.* [26].

6 Direct target of Cap1 according to Znaidi *et al.* [26].

Another interesting feature of the response to compound **2** was the induction of several multidrug transporter genes; namely *MDR1*, *SNQ2* and *CDR1*. *MDR1* showed particularly strong up-regulation (up to 43-fold at 60 min) and is known to be induced in response to oxidative stress [27], as well as by a hyperactive *CAP1* allele [24] consistent, again, with compound **2** triggering oxidative stress in *C. albicans*. However, it cannot be excluded that up-regulation of these multidrug transporter genes in response to compound **2** reflects a general drug response.

### A *Candida albicans cap1* Mutant Defective for Oxidative Stress Responses shows Hypersensitivity to Fused Mannich Ketones

**Figure 4:**
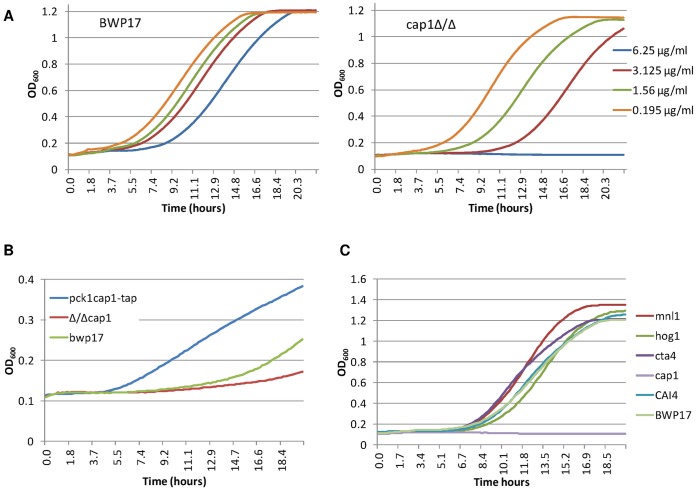
Growth kinetics in 96-well microtiter plates of *C. albicans* strains exposed to various concentrations of compound 2. **A.** Representative growth kinetics of BWP17 and *cap1Δ/Δ* strains exposed to decreasing concentrations of compound 2 in SD minimal medium. **B.** Representative growth kinetics of BWP17, *cap1Δ/Δ* and *CAP1* overexpression strains in inducible medium (YNB-casa) exposed to 12.5 µg/mL compound 2. **C.** Representative growth kinetics of several transcription factor mutants and parent strains in SD minimal medium exposed to 6.25 µg/mL compound 2.

Inactivation of the *CAP1* gene in *C. albicans* results in hypersensitivity to oxidative stress [24]. Hence, we compared the sensitivity of a wild-type strain and a *cap1▵/▵* mutant to compound **2**. The MIC_90_ of the *cap1▵/▵* strain was 4-fold lower than that of the wild-type strain (data not shown). Growth of the *cap1▵/▵* strain in microtiter plate was completely abolished at 6.25 µg/mL of compound **2** while the wild type strain showed a delayed but efficient growth compared to untreated cells (Fig. 4A). Moreover, the *cap1▵/▵* strain was more sensitive to lower concentrations of compound **2**, as indicated by a strong growth delay (Fig. 4A). In contrast, over-expression of *CAP1* resulted in an increase of the growth rate, compared to control and *cap1*▵/▵ strains, when exposed to compound **2** under inducible conditions (Fig. 4B). Taken together, these results indicated that a functional Cap1 regulon contributed to *C. albicans* survival upon exposure to FMKs.

### *Candida albicans* Mutants Defective for Non-oxidative Stress Responses do not show Hypersensitivity to Fused Mannich Ketones

In order to assess whether compound **2** triggered additional stress responses, we tested the sensitivity to this FMK of several *C. albicans* strains with null mutations in genes encoding signaling components involved in the response to a variety of stresses: *HOG1* encoding a MAP kinase involved in the response to osmotic stress and, to some extent, oxidative stress [28], [29]; *MNL1* encoding a transcription factor involved in the response to weak acid stress [30]; and *CTA4* encoding a transcription factor involved in the response to nitrosative stress [31]. Results presented in Fig. 4C showed that, while growth of the *cap1▵/▵* strain is inhibited at 6.25µg/mL compound **2**, this was not the case for the *hog1▵/▵*, *mnl1▵/▵* and *cta4▵/▵* mutants. Similar results were obtained when the mutant strains were exposed to H_2_O_2_–with the *mnl1▵/▵* and *cta4▵/▵* strains behaving like the wild-type strain, the *cap1▵/▵* strain showing high sensitivity to H_2_O_2_ and the *hog1▵/▵* strain showing moderate sensitivity to H_2_O_2_ (data not shown). In contrast, only the *hog1▵/▵* mutant showed sensitivity to osmotic stress (data not shown). Again, these results indicated that compound **2** elicited a specific oxidative stress in *C. albicans* and that survival of *C. albicans* after exposure to compound **2** was specifically dependent upon a functional Cap1 regulon.

## Discussion

We have shown that a subset of fused Mannich ketones have antifungal activity against pathogenic yeasts. In particular, FMKs with a five-membered saturated ring (compounds **1–4**) showed the best potency toward *C. albicans* and the closely related *C. parapsilosis* and *C. krusei* while having reduced potency toward *C. glabrata*. Among them, only compound **2** was effective against *Aspergillus sp*. Interestingly, these four FMKs (**1**–**4**) showed moderate toxicity to mammalian cells, and our results with compound **2** indicated that it also had moderate toxicity in mice. In this study, we have additionally investigated the effect of one of these FMKs, compound **2**, on *C. albicans* by transcript profiling and shown that it elicited a strong oxidative stress-like transcriptional response. Notably, many of the genes whose transcription was increased upon compound **2** treatment were regulated by the *C. albicans* oxidative stress-response regulator Cap1p, and we showed that inactivation of the *CAP1* gene increased the sensitivity of *C. albicans* cells to compound **2**. Taken together, these data have suggested that generation of oxidative stress is an important component of the antifungal activity of compound **2** and, more generally, FMKs.

Whether FMKs directly or indirectly generate an intracellular oxidative stress has not been determined. Dimmock *et al.* [32] have proposed that Mannich bases of unsaturated ketones can be considered alkylating agents reacting with low molecular-weight and protein-associated thiols through Michael addition. In particular, they have investigated compound NC1175, a conjugated styryl ketone exhibiting a wide-spectrum activity against pathogenic fungi, and suggested that its activity is probably based on the inhibition of H^+^-ATPase-mediated proton pumping [33]. Alternatively, it has been proposed that Mannich ketone-induced thiol depletion could cause oxidative stress leading to oxidative damage of the cell and production of reactive oxygen species [34]. The Mannich ketones that have been investigated in the present study can be classified as fused cyclic Mannich ketones. While the unsaturated Mannich ketones contain two alkylation sites for the potential thiol nucleophiles (*i.e.*, the C = C bond and a latent alkylation site), FMKs have only one latent alkylation site. Indeed, the amine group can be considered a latent alkylation site, since its 1,2-elimination under physiological conditions affords a very reactive vinyl ketone that can react with thiols according to Dimmock *et al.* [32]. The observation that aminoalcohols **22** and **23** devoid of this latent alkylation site did not elicit antifungal activity suggests that FMKs could directly trigger oxidative stress. The implication of oxidative stress into the prevailing mechanism of action of FMKs is further supported by the strong correlation between FMKs’ antifungal potency and their LUMO energies in QSAR studies. Specifically, the formation of radical anions from FMKs initiating ROS formation through redox cycling would be facilitated by the low LUMO energies of these compounds. A similar mode of action has been proposed for 7-chlorotetrazolo[5,1-c]benzo [1], [2], [4]triazine (CTBT), a compound with chemosensitizing activity in yeasts [35]. Specifically, transcript profiling of *S. cerevisiae* exposed to CTBT revealed the induction of oxidant- and stress-response defense genes as well as nuclear translocation of Yap1p, a transcriptional regulator with functions similar to *C. albicans* Cap1p [35].

While QSAR results suggested that FMKs antifungal activity is a direct consequence of their ability to trigger oxidative stress, it should be taken into consideration that other antifungals with defined modes of action have been shown to elicit oxidative stress, in possibly indirect ways. This is the case for benomyl that targets microtubules. Benomyl triggers transcriptional responses similar to those associated with oxidative stress inducers such as H_2_O_2_ in *C. albicans* [25], [36], [37] and oxidative stress responses in *S. cerevisiae* [38]. In this respect, we have observed a striking overlap between the 56 genes identified in our study as up-regulated in response to compound **2** and genes identified as up-regulated in response to benomyl by Karababa *et al.* [37] and Znaidi *et al.* (Table 4, [26]). However, the mechanisms by which benomyl elicits oxidative stress are not known. Polyenes, azoles and echinocandins have also been shown to elicit an oxidative stress response, although to an apparently much lower extent than compound **2.** While amphotericin B has been known to bring forth oxidative damage [39], [40], the amphotericin B-dependent transcriptional and translational responses of oxidative stress response genes are considerably weaker than those we observed upon treatment with compound **2** [41], [42]. Moreover, most of the oxidative stress-response genes induced upon amphotericin B treatment are not Cap1 target genes [26], and no change in *CAP1* expression or levels of the Cap1 protein are associated with amphotericin B treatment. In *S. cerevisiae, YAP1* does not influence the susceptibility to amphotericin B [43]. Nuclear translocation of Cap1 in response to the echinocandin caspofungin has been reported along with the induction of two Cap1-regulated genes, *GLR1* and *SOD2* [44]. However, transcript profiling of caspofungin-treated *C. albicans* demonstrated repression of *CAP1* and no up-regulation of oxidative stress genes [42], suggesting that very high concentrations of caspofungin, as those used by Kelly et al. [44], are required to observe oxidative stress. Finally, exposure to ketoconazole does not trigger overexpression of stress genes [42] while fluconazole can induce 2 Cap1-dependent oxidative stress genes, *TRR1* and *GRE2* [45]. Thus, massive Cap1-dependent oxidative stress responses, as those observed upon treatment of *C. albicans* by compound **2**, do not appear to be a generic trait of most antifungal agents, providing support for the hypothesis that FMKs directly trigger oxidative stress that explains their antifungal activity. Nevertheless, the precise mode of antifungal action of FMKs and whether their triggering of oxidative damage is direct or indirect will require further investigation.

Finally, our results outline a possible chemosensitizing potential for Mannich ketones, as QSAR and transcript profiling against *C. albicans* have been very similar to those observed with the chemosensitizing agent CTBT on *S. cerevisiae* [35]. Chemosensitizing approaches are particularly promising to enhance the efficacy of clinically-relevant antifungals, allowing dose and side effects reduction. Compounds with redox potential and targeting the oxidative stress response, such as compound **2**, have been described as chemosensitizing agents and proven to enhance antifungal efficacy of azole and amphotericin B against pathogenic yeasts including *C. albicans* [43], [46]. Thus, the chemosensitizing potential of Mannich ketones should be explored in the future.

## Materials and Methods

### Strains, Media and Antifungal Agents

Strains are listed in Table 3. To construct the *CAP1* overexpressing strain (CEC3490), the *CAP1* gene was cloned in the CIp10-PCK1p-GTW-TAPtag vector and the resulting plasmids were introduced at the *C. albicans* RPS1 locus as described in Cabral *et al.* [47]. This construction placed the *CAP1* gene under the casaminoacids inducible PCK1 promoter. International standard strains, *C. albicans* strains, *Saccharomyces sp.* and *Aspergillus sp.* strains from clinical samples were cultivated in Sabouraud medium (OXOID Ltd., England), RPMI-1640 medium (Sigma-Aldrich, Hungary) buffered to pH 7.0 with 0.165 M MOPS (Sigma-Aldrich, Hungary) or SD minimal medium (0.67% yeast nitrogen base without amino acids [Difco] plus 2% glucose) supplemented with uridine (40 mg/l), arginine (20 mg/l), or histidine (20 mg/l), when needed. *C. albicans* overexpression strains were cultivated in SD minimal medium (uninduced condition) or YNB-casa (0.67% yeast nitrogen base without amino acids [Difco] plus 2% casaminoacids; induced condition). Amphotericin B (Fungizone, Bristol-Myers Squibb, Epernon, France) was used as an antifungal standard. Fused Mannich ketones (compounds **1–21**) and the corresponding aminoalcohols (compounds **22–23**) were synthesized as described previously [8], [9]. Some have been known compounds prepared according to the literature [48], [49], [50], [51], [52], [53], [54], [55], [56], [57], [58], [59], [60]. All data of the test compounds were in accordance with the compounds published in the references above. Minimum inhibitory concentration (MIC) values of standard and synthesized agents were determined using the macro-tube dilution method [13]. Briefly, the test compounds were dissolved in dimethyl sulfoxide (DMSO) and diluted in RPMI. In the first tube there was 100 µg/mL test material in 5% (v/v) DMSO. From this tube we made double dilution series. The inoculum concentration was 10^4^ cells/mL. The tubes were incubated for 48 h at 30°C. They were checked by naked eye and subcultures were made from the tubes on Sabouraud medium agar. The colony forming unit (CFU) values were determined after incubation for 48 hours at 30°C. MIC_90_ stands for the drug concentration that yielded a 10-fold reduction in CFUs relative to an untreated control. MIC_90_s were determined three times for each compound and strain. Other technical details have been given in a previous publication [13].

### In vivo Toxicity Testing

Cytotoxicity tests in HeLa cells were performed as described previously [9]. Animal experiments were approved by the Animal Ethics Committee of Pécs University (Permission Number: BA/02/2000-1/2007). The work regulation of this Committee is based on Good Laboratory Practice (GLP) and harmonized with Directive 2010/63/EU on the protection of animals. For the determination of median lethal dose (LD_50_), inbred male and female BALB/c mice, weighing 18–22 g each, were provided with normal mice chow and water *ad libitum.* All animals were allowed to acclimate for at least 5 days prior to the first treatment. The 160 mice were randomly divided into 8 groups. Compound **2** was resuspended in physiologic saline solution at the final concentration of 1, 2, 4, 8, 10, 12, 16 and 20 mg/mL and 1 mL of each solution was injected intraperitoneally into 20 mice each. The animals were under control for 7 days and the death rate was determined. The LD_50_ value was determined from the dose–effect curve by Lichfield-Wilcoxon graphic methods [19], [20].

### QSAR Calculations

QSAR analysis was done using the BioMedCAChe 6.1 program for Windows (Fujitsu, Beaverton, OR). Structures were preoptimized using augmented MM3 parameters followed by semiempirical PM/3 optimization. A total of 32 descriptors were calculated for each compound. The empirical MICs were expressed in molality and converted to pMIC (negative logarithm of MIC) values. Only compounds with an MIC <200 mg/l were used in the QSAR analysis. The best QSAR equations with three and two descriptors were selected by multiple linear regressions via the Project Leader module of BioMedCAChe. Model validations were done by randomization of pMIC and leaving out 10% of the compounds.

### Microarray Experiments

Gene expression analysis of the *C. albicans* sequenced laboratory strain SC5314 was performed by comparing planktonic cells with and without exposure to compound **2**. An exponentially growing *C. albicans* culture in SD medium at 30°C was split into two flasks, one exposed to MIC_90_ concentration of compound **2** (6.25 µg/mL in water), the other to the same volume of water. Samples were collected after 30 and 60 min for transcript profiling. Total RNA was isolated using an RNeasy minikit (Qiagen, Courtaboeuf, France) according to the manufacturer’s instructions. The concentration, purity, and integrity of the isolated RNA were evaluated using a Nanodrop spectrophotometer (Thermo Fisher, Illkirch, France) and an Agilent 2100 Bioanalyzer (Agilent Technologies, Waldbronn, Germany). cDNA synthesis, labeling and hybridization on *C. albicans* oligonucleotide microarrays (Eurogentec, Liege, Belgium) were performed as described in Rossignol *et al.* [14]. Sample comparisons at 30 and 60 min were performed on two biological replicates, and each biological replicate was subjected to technical replicates with dye swap.

### Microarray Analysis

Microarray scans were performed with a GenePix 4000 A scanner using GenePix 5 software and analyses were performed using GeneSpring GX software (Agilent Technologies, Massy, France). Data normalization was performed with the LOWESS method, and the statistical analysis with the t-test from GeneSpring. We used the September 2011 annotation from the Candida Genome Database [23]. Some oligonucleotides on the microarrays did not match a gene in the current version of CGD as some genes have been removed from CGD or coordinates have been refined. Data for these oligonucleotides were not analyzed further. Genes regulated by at least 1.5-fold with P<0.05 were considered significant. Microarray data have been deposited at ArrayExpress under accession number E-MEXP-3534. Normalized data are available in Table S1 in the supplemental material. Gene ontology analyses were performed using tools available at the Candida Genome Database, with p-values calculated by GO Term Finder [22]. GO TermFinder calculates a *P*-value using the hypergeometric distribution:
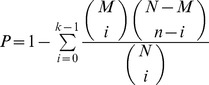
where N is the total number of genes in the background distribution, M is the number of genes within this distribution that are annotated to the node of interest, n is the list size of the genes of interest, and k is the number of genes within this list annotated to the node.

### Growth Kinetics

Strains were inoculated at a final OD_600_ = 0.05 in 100 µL and were grown in 96-well plates at 30°C for 24 h. Growth was monitored every 20 minutes using a microplate reader (TECAN Sunrise). For growth kinetics comparison of wild type and *cap1* deleted strains, growth curves were performed in triplicate in independent experiments in SD minimal medium complemented with various concentrations of compound **2** ranging from 25 µg/mL to 0.0975 µg/mL. For *CAP1* overexpression experiments, growth curves were performed in duplicates in independent experiments in SD minimal medium (non-induction condition) and in YNB-casa (induction condition) with various concentrations of compound **2** ranging from 25 µg/mL to 0.0975 µg/mL. For comparison between transcription factor mutants and parent strains experiments, growth curves were obtained in duplicates from independent experiments in SD minimal medium with various concentrations of compound **2** ranging from 25 µg/mL to 0.0975 µg/mL, or H_2_O_2_ ranging from 4 mM to 15.62 µM. For all these experiments, OD_600_ readings at final point were also recorded in two independent experiments for validation.

## Supporting Information

Table S1 **Microarray Gene expression data**. Normalized expression data for all ORFs with fold change ratios and p-values at 30 min and 60 min as described in materials and methods section.
(XLS)
